# Combined immunization with inactivated vaccine reduces the dose of live *B. abortus* A19 vaccine

**DOI:** 10.1186/s12917-022-03229-0

**Published:** 2022-04-02

**Authors:** Chuan-Yu He, Yu-Zhuo Zhang, Meng-Zhi Liu, Hai-Long Zhao, Li-Song Ren, Bao-Shan Liu, Sun He, Ze-Liang Chen

**Affiliations:** 1 Key Laboratory of Livestock Infectious Diseases, Ministry of Education, Shenyang Agricultural University, Liaoning Province 110866 Shenyang, P. R. China; 2 TECON Biological Co., Ltd, 221 Lantian Road, 830000 Urumqi, P. R. China; 3 College of Animal Husbandry and Veterinary Medicine, Shenyang Agricultural University, 120 Dongling Road, Shenyang, 110166 China

**Keywords:** Brucellosis, Combined immunization, Live vaccine, Inactivated vaccine

## Abstract

**Background:**

*Brucella spp*. is an important zoonotic pathogen responsible for brucellosis in humans and animals. *Brucella abortus* A19 strain is a widespread vaccine in China. However, it has a drawback of residual virulence in animals and humans.

**Methods:**

In this study, the BALB/c mice were inoculated with either 100 μL PBS(control group, C group), 10^9^ CFU/mL inactivated *B. abortus* A19 strain (I group), 10^5^ CFU/mL (low-dose group, L group) 10^6^ CFU/mL live *B. abortus* A19 strain (high-dose group, H group), or 10^5^ CFU/mL live *B. abortus* A19 strain combined with 10^9^ CFU/mL inactivated *B. abortus* A19 strain (LI group). Mice were challenged with *B. abortus* strain 2308 at 7 week post vaccination. Subsequently, the immune and protective efficacy of the vaccines were evaluated by measuring splenic bacterial burden, spleen weight, serum IgG, interferon-gamma (IFN-γ), interleukin-4 (IL-4) percentage of CD4 + and CD8 + T cells of mice via bacterial isolation, weighing, ELISA and flow cytometry, respectively.

**Results:**

The splenic bacterial burden and spleen weight of the mice in group LI were mostly equivalent to the mice of group H. Moreover, *Brucella*-specific serum IgG, IFN-γ, IL-4, and the percentage of CD4^+^ and CD8^+^ T cells of the LI group mice were similar to those of the H group. In the subsequent challenge test, both vaccines conferred protective immunity to wild-type (WT) 2308 strain. In addition, the levels of IL-4 and IFN-γ, CD4^+^ and CD8^+^ T cells in these mice were similar to those of the mice in the H group.

**Conclusions:**

Combined immunization with low dose live vaccine and inactivated vaccine allowed to reduce the live *B. abortus* A19 vaccine, dose with an equivalent protection of the high-dose live vaccine.

**Supplementary Information:**

The online version contains supplementary material available at 10.1186/s12917-022-03229-0.

## Background

*Brucella spp*. are gram-negative bacteria responsible for brucellosis. This bacterial infection has caused serious economic losses in livestock and harm to human health [1, 2]. Disease caused by *Brucella spp*. can be either acute or chronic. One of the consequences of infection is abortion [3].

Vaccination is critical to preventing and controlling brucellosis in humans and livestock [4, 5]. Currently, the vaccines used in China include attenuated *Brucella abortus* strain A19 (an erythritol-resistant homologous of *B. abortus* strain S19, able to grow in the presence of erythritol), *Brucella melitensis* strain M5, and *Brucella suis* strain S2 strain. *B. abortus* S19 has been widely used to prevent brucellosis in cattle [6–8]. The bacterium was isolated from a cattle farm in New Jersey in 1923, becoming a weakened vaccine strain after more than one year of passage in the laboratory [9]. Although the *B. abortus* A19 vaccine is commercially available for cattle and effective in protecting cattle when challenged with WT 2308 *B. abortus*, it has residual virulence. This is a risk for both animals and humans [6, 10], it *can cause* infection during vaccination [6, 11].

Therefore, vaccines with low residual virulence and high protective efficacy are needed to overcome these limitations. Up to now, experimental challenge studies with live *Brucella* strains have mainly focused on a marker vaccine, which is designed by knocking out the virulence genes from the parental vaccine strains [12, 13]. However, developing new live vaccine strains based on this strategy is expensive and time-consuming [4]. Combination immunization using existing attenuated and inactivated vaccines may be a better way. Therefore, in this study, we attempted to combine the low-dose live *B. abortus* A19 vaccine and inactivated *B. abortus* A19 vaccine to examine its efficacy compared to that of the high-dose live *B. abortus* A19 vaccine, with the objective of decreasing the A19 dose to reduce the infection risk in both animals and humans.

## Results

### Spleen weight and bacterial burden of inoculated mice

**Fig. 1:**
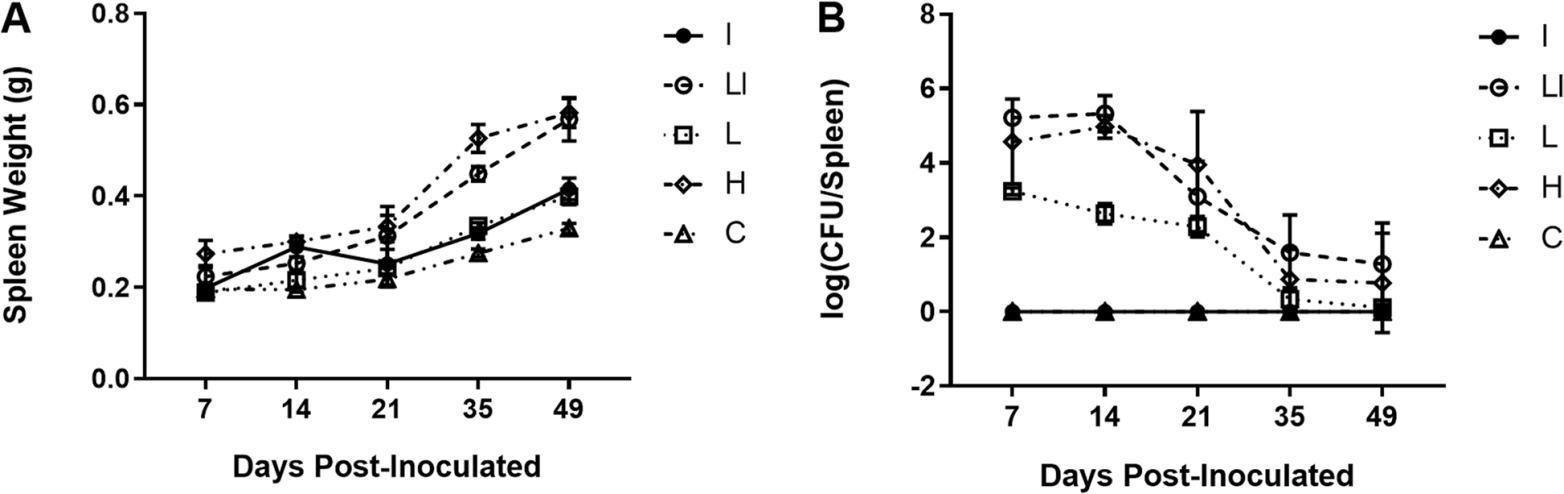
Spleen weight and bacterial burden of inoculated mice. BALB/c mice were inoculated intraperitoneally with inactivated, low-dose live + inactivated, low dose live, high dose live *B. abortus* A19, or sterilized saline. Low-dose live- + -inactivated-inoculated mice show reduced splenic bacterial burden during the chronic phase of infection, similar to the mice of the high-dose live vaccine but lower than mice of the other groups. At 1, 2, 3, 5, and 7 weeks post-inoculation, mice were euthanized and the spleens were weighed (**A**) and were used to determine the bacterial burden (**B**). Data are presented as the mean ± the standard error of the mean (*n* = 6)

A gradual increase in the spleen weight of the inoculated mice was observed from 7 to 49 days post-inoculation (Fig. 1A). However, the highest spleen weight for both H and LI groups was observed 49 days (≈ 0.50 g). However, there was no significant difference between the two groups. The difference in spleen weight between the two groups occurred at 14 and 35 days post infection (*P* < 0.05), and there was no difference with the other three sampling stages (*P* > 0.05).

Bacterial burden indicates that *B. abortus* replicates successfully in spleen cells. On the day 7, 14, and 21 after immunization, there were significant differences in the splenic bacterial loads between groups I and C and the other groups (*P* < 0.05). At 14 days from vaccination, there was a significant difference in the number of bacteria in the spleen between the L and group H groups (*P* < 0.05). The highest bacterial burden in the LI group occurred at 14 days post-vaccination, followed by a significant decrease (Fig. 1B). However, there was no significant difference with group H (*P* > 0.05). Although the spleen bacterial burden of the LI group was slightly higher than that of group H, their overall trend was similar.

### IgG and cytokines of the inoculated mice

**Fig. 2:**
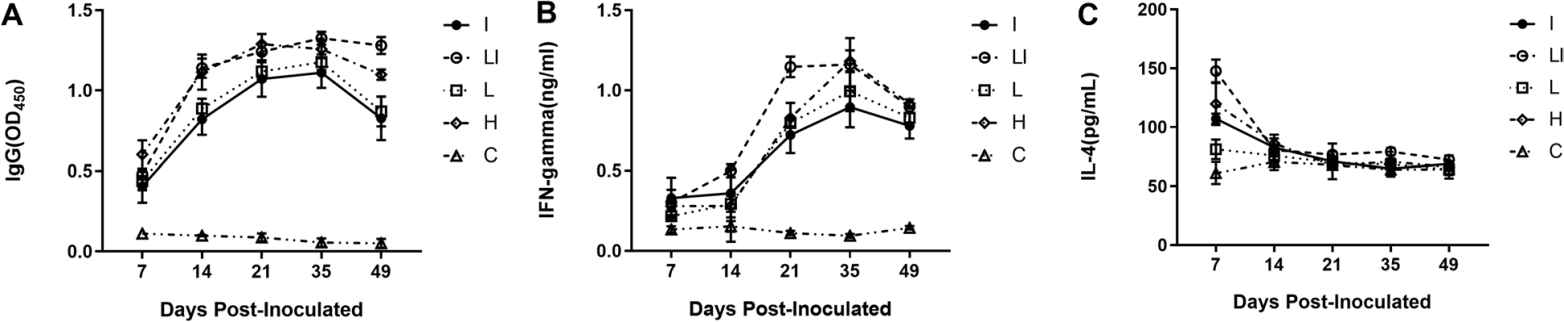
Antibody and cytokine quantification of the vaccinated mice. Mouse serum was harvested at 1, 2, 3, 5, and 7 weeks post-inoculation from mice infected with high-dose live, low-dose live, low-dose live + inactivated, and inactivated *Brucella*, and a control group. Amounts of *Brucella*-specific IgG (**A**), interferon-gamma (IFN-γ) (**B**) and interleukin-4 (IL-4) (**C**) were quantified by enzyme-linked immunosorbent assays (ELISA). Each data point (*n* = 6) is the mean ± the standard error of the mean

Serum was harvested from control mice and inoculated mice at 1, 2, 3, 5, and 7 weeks post-inoculation (wpi) and used to measure immunoglobulin G (IgG) levels specific to *B. abortus*, as well as inerferon gamma (IFN-γ) and interleukin-4 (IL-4) by ELISA. *Brucella*-specific IgG was higher at 2 and 7 weeks in both the H and LI groups than that in the mice of group I, L and the control group (*P* < 0.05) (Fig. 2A).

Furthermore, the IFN-γ levels of the H and LI groups increased over the first five weeks of inoculation and decreased at the end of the 7 wpi (Fig. 2B). The concentration of IFN-γ at 3 wpi for group LI was higher than that of group H (*P* < 0.05). This indicates that the combined use of live and inactivated vaccines can increase the secretion of IFN-γ.

IL-4 level in all groups peaked at the first wpi. However, the IL-4 level in the LI group was higher than that in groups I, L, and C (*P* < 0.05) (Fig. 2C), except for group H. Following one wpi, groups H and LI showed a gradual downward trend up to 3 wpi. In contrast, no significant differences in the IL-4 levels in the various groups were observed. However, five weeks after immunization, IL-4 levels in the live vaccine group were significantly higher than those in the inactivated vaccine and control groups (*P* < 0.05), suggesting that the live vaccine significantly increased IL-4 production. Cumulatively, these results show that the low-dose live + inactivated vaccines could elicit an equivalent antibody and cytokine response to the live vaccine in BALB/c mice. Furthermore, the low-dose live + inactivated vaccine may elicit an earlier and faster immune response.

### CD4 + and CD8 + T cells of the inoculated mice

**Fig. 3:**
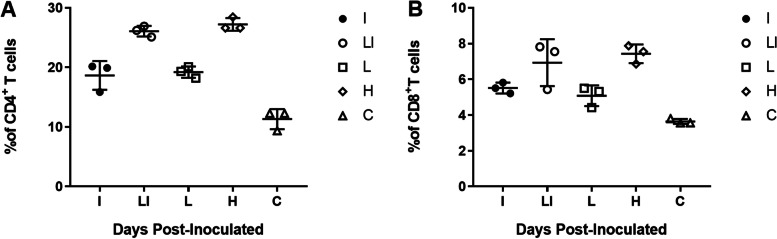
CD4^+^ and CD8^+^ T cells quantification of the vaccinated mice. Intraperitoneal vaccination stimulates the production of CD4^+^ and CD8^+^ T cells in BALB/c mice. Production of CD4^+^ and CD8^+^ T cells was evaluated by flow cytometry

Flow cytometry analysis of the plasma blood of mice at 2 wpi was used to ascertain the amount of CD4^+^ and CD8^+^T cells induced by inoculation (Fig. 3). Results indicated that the number of CD4^+^T cells was higher than that of CD8^+^ T cells in LI (*P* < 0.05) (Fig. 3). The levels of CD4^+^ in groups LI and H were significantly higher than those in the other groups (*P* < 0.05) (Fig. 3A). However, the expression of CD8^+^ in group H was markedly higher than that in group L (*P* < 0.05), but there was no difference with group LI (*P* > 0.05) (Fig. 3B).

### Protective effects against the *B. abortus* strain 2308 challenge

**Fig. 4:**
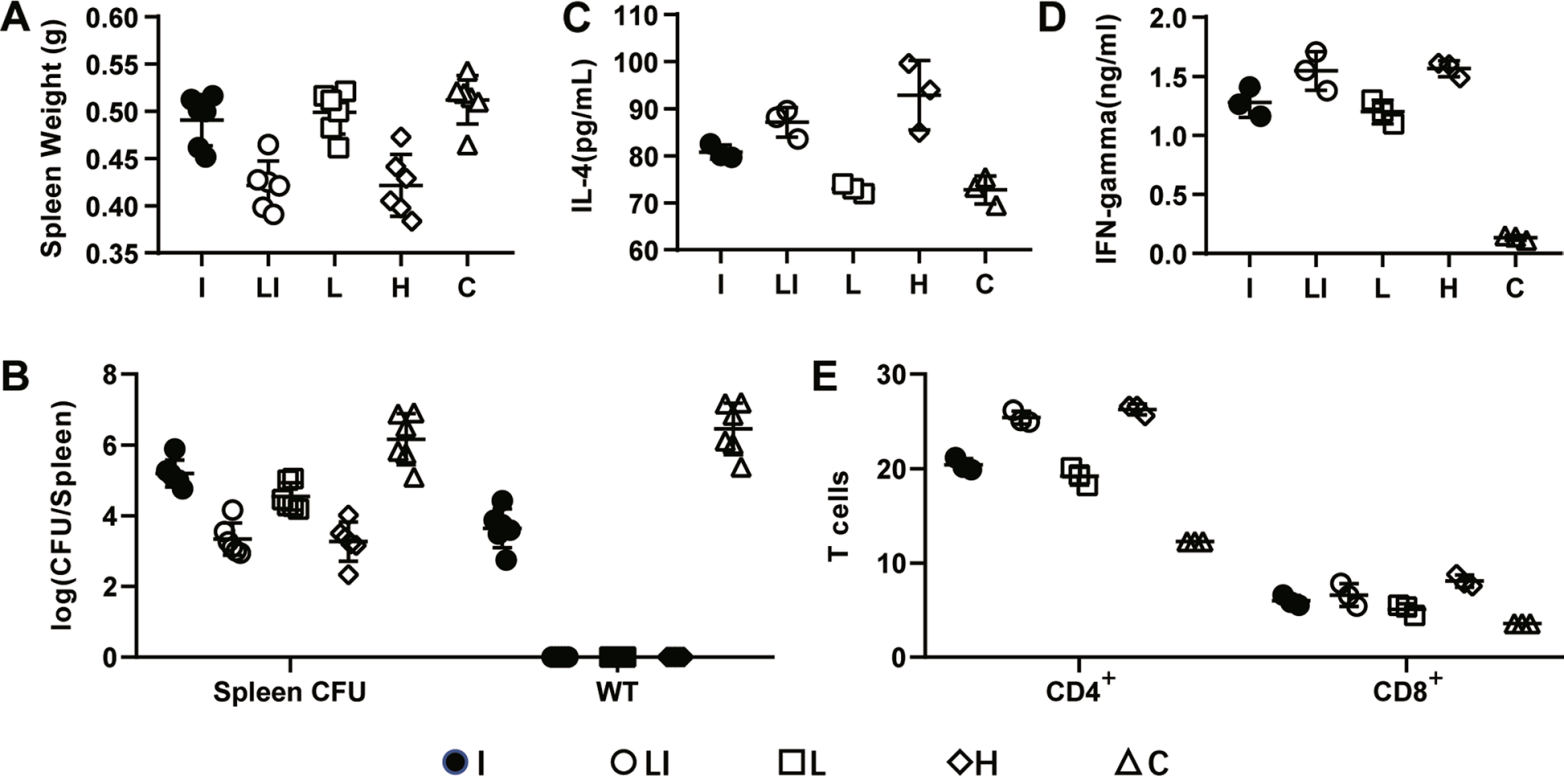
Analysis of the vaccinated mice after the challenge with WT 2308 *Brucella* seven weeks post-vaccination. Data are presented as the mean ± SD (*n* = 6)

Mice in groups I, L, and C showed considerable splenomegaly with a mean spleen weight of 0.53 ± 0.08 g, 0.499 ± 0.023 g and 0.512 ± 0.026 g, respcecively, at three weeks post-challenge (WPC) (Additional file 1, Fig. 4A). Mice in the LI and H groups showed a significantly lower spleen weight than above groups, with 0.421 ± 0.026 g and 0.421 ± 0.033 g, respectively (*P* < 0.05). However, the spleen weight of the mice in the LI and H groups was not difference(*p* > 0.05). Reduced splenomegaly corresponded with a reduction in bacterial burden. The spleen CFU changed with the spleen weight. Mice in groups H and LI had 3 logs less splenic bacteria than control mice at three wpc. In contrast, mice immunized with the inactivated vaccine had 1 log less splenic bacteria than control mice(Fig. 4B).

We subsequently assessed whether control, live, low-dose live + inactivated, and inactivated groups showed differences in WT, IL-4, IFN-γ, CD4^+^ T cell, and CD8^+^ T cell production after WT 2308 challenge. No WT was isolated in mice of the groups LI, L and H, which is significantly lower than in groups I and C (*P* < 0.05) (Fig. 4B). IL-4 levels in the H and LI groups were similar and slightly higher than those in the control group (*P* < 0.05) (Fig. 4C). Regarding IFN-γ, levels in the groups H and LI were higher than those in groups L and C (*P* < 0.05) and equal to the group I (Fig. 4D). The number of CD4^+^ T cells was higher than that of CD8^+^ T cells in the LI and H groups. The CD4^+^ and CD8^+^ T cells of the two groups had no significant difference (*P* > 0.05). However, they were higher in group H compared with the groups I and C (*P* < 0.05) (Fig. 4E).

## Discussion

This study aimed to design a new method for vaccine immunization, a safer alternative to conventional vaccines, which would provide sufficient protection against *Brucella*. Vaccination indicators and the protective effect were measured in the inoculated mice. The results showed that immunization of mice with a low-dose live + inactivated vaccine could induce a strong humoral and cellular immunity response and maintain a high protective efficacy similar to that of the high-dose live vaccine. This suggests that the combination vaccination of low-dose live and inactivated vaccine is a suitable immuniszation method to reduce live bacteria.

Existing research suggests that using a live vaccine is one of the most effective ways to prevent and control brucellosis [14, 15], although conventional brucellosis vaccines are only approximately 70% effective [6, 14]. However, most licensed vaccines have residual toxicity and diagnosis interference [16, 17]. For example, *B. abortus* A19 used in many countries [18, 19] can lead to abortion in 3.2% of pregnant cattle after vaccination and is generally more pathogenic than its competitors [20]. Therefore, the dose of live vaccine administered to pregnant females and breeding males is reduced. Even worse, the vaccine can infect people and cause them to become ill [21, 22]. Therefore, the need arose for an alternative vaccine to be developed that would overcome these issues. However, producing an alternative vaccine for brucellosis has been challenging in recent years[5]. Making the best use of existing vaccines is a solution before a better one becomes available.

The immunization with low vaccine doses is infeasible as it induces a weak antibody response [18]. Another option is the combination of existing vaccines. In this study, we attempted to use a combination of low-dose live and inactivated *B. abortus* A19. The live vaccine induces cellular immunity while the inactivated vaccine induces humoral immunity to make up for the lost immunogenicity and protective ability [23]. The combination of the two may have a good preventive effect.

The spleen of mice immunized with low dose live + inactivated vaccines was enlarged compared with that of mice vaccinated with the high-dose live vaccine, but the spleen weight was slightly reduced and spleen bacterial load was fairly increased (Fig. 1B). This suggests that the two inoculation methods might elicit similar host immune responses. For testing this hypothesis, serum levels of specific cytokines and antibodies important in countering *Brucella* infection were measured. Results showed no pronounced differences in IL-4 and IFN-γ levels between groups H and LI over the 7 weeks. The peak in Brucella-specific IgG response was almost the same in both groups 3 wpi, suggesting that both inoculation methods may induce a similar robust humoral response.

The ideal vaccination must induce high protective efficacy [24]. Therefore, we evaluated the effectiveness of immunization in BALB/c mice. The vaccinated mice in group LI showed identical inflammation and bacterial load in the spleen compared with group H when challenged with WT 2308 *B. abortus*. This demonstrates that the LI and H immunization methods were similar in their ability to conteract splenic colonization by *Brucella*.

IFN-γ is a critical Th1-type immune cytokine in the immune response against *Brucella* and is required for macrophage bactericidal activity [25, 26]. Although BALB/c mice are mainly protected by Th2-type responses, the Th1-type pathway can also plays a rol against bacterial infections. Therefore, we assessed antigen-specific production of Th1-type cytokines (IFN-γ) and Th2-type cytokines (IL-4) in serum from the LI and H groups. Similar levels of IFN-γ and IL-4 were measured in the LI and L groups after WT challenge, indicating that both vaccines elicited a combination of Th1- and Th2-type immune responses. However, the high IFN-γ levels in the two groups suggest that Th1-mediated protection is the favored response.

Although CD4 + T cells are also responsible for the Th2-type of immune response, they mainly mediate the Th1-type, which is critical for removing *Brucella* from infected cells [27, 28]. The number of CD4^+^ T cells can be directly related to the level of the Th1-type response. On the other hand, CD8^+^ T cells mainly mediate the naive T cell (TN) immune response [29, 30]. The higher the number of CD4^+^/CD8^+^ T cells, the more robust the Th1-type response [29–32]. In this study, immunization with the LI induced more CD4^+^ than CD8^+^ T cells, corresponding to the experimental groups immunized with the live vaccine. This result is also consistent with those of cytokine tests.

The results showed that both the high-dose live vaccine and the low-dose live + inactivated vaccines elicit similar host immune responses and protection against the *B. abortus* strain 2308. Comparing the immune effects of group H and group L, we found that the combined immunization mainly improved the levels of IgG and T cells in the L group.

## Conclusions

This study showed that the combination immunization with inactivated and low-dose live vaccines could induce a strong humoral and cellular immunity response, generatin a high protective efficacy similar to that of the high-dose live vaccine. However, there are no reports showing that reducing the dose of the brucellosis vaccine is safe. However, reducing the dose of a live vaccine may reduce people's exposure during animal immunization and still have a positive effect on preventing human vaccine infection. However, whether a smaller dose of live vaccines can exert the same influence and whether it shows the same immune effect on cattle require further experiments.

## Methods

### Mice and ethics statement

The research was approved by the animal welfare and ethics committee of TECON Biology Co., Ltd. (TBCA20-014). 8-week-old female BALB/c mice were obtained from Xinjiang Medical University. All animals were handled following the Experimental Animal Regulation Ordinances defined by the Chinese National Science and Technology Commission. Humane care and healthful conditions were provided for the animals. All individuals who used animals had received instruction in experimental methods and the care, maintenance, and handling of mice under the committee’s supervision. All animal experiments were performed in accordance with ARRIVE guidelines.

### Bacterial strains

For this study, we used the wild-type *B. abortus* strain 2308 and the *B. abortus strain A19.* Bacterial strains were grown for three days on *Brucella* Agar(BD BBL™) at 37 ℃ before being suspended in sterile phosphate-buffered saline (PBS; pH7.2), to a concentration of 10^6^ and 10^5^ CFU/mL. The *B. abortus A19* strain was diluted to a 1 × 10^9^ CFU/mL concentration in PBS for inactivation. Culture and maintenance of bacterial strains were performed as previously described [33–35].

### Vaccination of mice

**Fig. 5:**
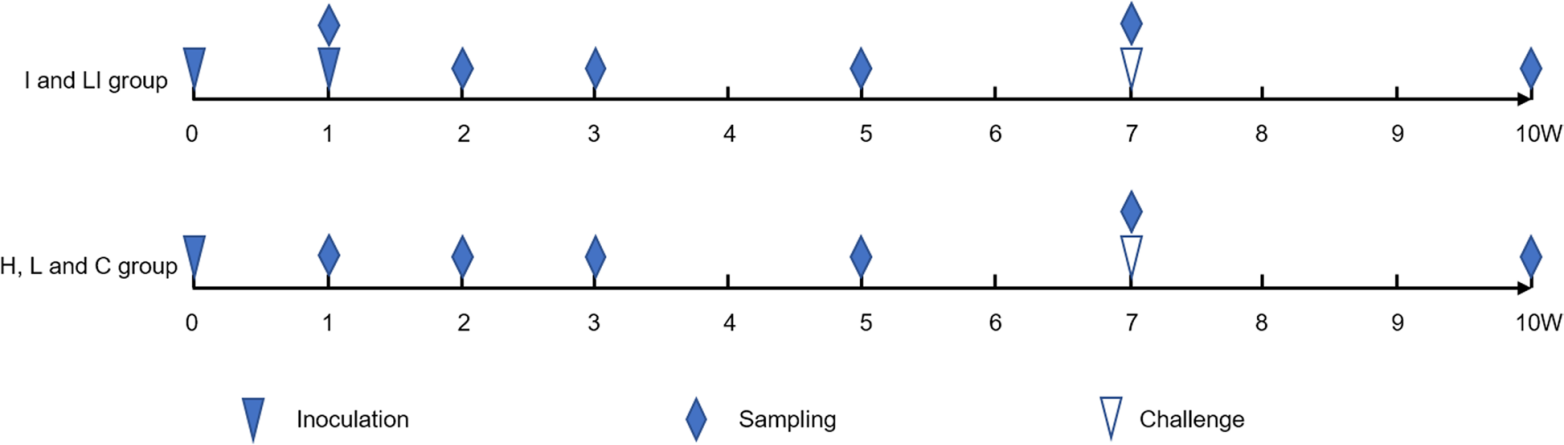
Timeline of inoculation, sampling, and challenge

BALB/c mice were randomly divided into five groups (*n* = 36 per group) for intraperitoneal (IP) vaccination. Each mouse in the first group (group H) was subjected to IP vaccination with 100 μL 10^6^ CFU/mL live *B. abortus A19*. Each mouse in the second group (group L) was subjected to IP vaccination with 100 μL 10^5^ CFU/mL live *B. abortus A19*. Each mouse in the third group (group LI) was subjected to IP vaccination with 100 μL 10^5^ CFU/mL live *B. abortus A19*, with a second round of IP vaccination with 100 μL 10^9^ CFU/mL inactivated *B. abortus* A19 (at 100 ℃for 30 min) after one week. Each mouse in the fourth group (group I) was subjected to IP vaccination twice with 100 μL 10^9^ CFU/mL inactivated *B. abortus* A19 with a 7-day interval (Fig. 5). Each mouse in the fifth group (group C) was subjected to IP injection with 100 μL sterile saline.

### Post-vaccination sample collection

Samples were collected from the vaccinated mice at 1, 2, 3, 5, and 7 weeks post the first inoculation. The animals were euthanized by CO_2_ asphyxiation before aseptic removal of spleens and blood collection via cardiac puncture for serum separation. Serum samples were subsequently stored at *-*20 ℃ until all the time points were completed.

### Spleen weight and bacterial burden

Spleens removed from the euthanized mice were weighed and splenic bacterial burden (CFU/spleen) was determined by homogenizing each spleen in 3 mL PBS. Serial dilutions were prepared for each sample and 100 µL was plated on *Brucella* Agar (BD BBL™).

### Detection of *Brucella*-specific antibody and determination of cytokine response

*Brucella*-specific IgG, IFN-γ, and IL-4 were measured using an enzyme-linked immunosorbent assay (ELISA). *Brucella* broth was used to culture *Brucella* abortus for three days, and PBS (OD600 = 1.8) was used to prepare a bacterial suspension. These cultures were then heat-killed at 80 ℃ for 1 h, cooled to room temperature, and treated with kanamycin (50 g/mL) and gentamycin (50 g/mL) to prevent bacterial growth. ELISA plates were coated with 100 μL heat-killed bacteria to determine *Brucella*-specific immunoglobulin concentration. Then IFN-γ and IL-4 titers were measured using the mouse IFN-γ ELISA kit (BD Biosciences, New Jersey, USA) in accordance with the manufacturer’s instructions. Absorbance was measured at 450 nm, with a 570 nm background correction, using a BioTek Epoch2c plate reader.

Two weeks after vaccination, 200 µL of mouse anticoagulated peripheral blood was drawn through the submandibular vein, and surface-labeled monoclonal antibodies CD4 FITC (BD Pharmingen™ FITC rat anti-mouse CD4) and CD8 PE (BD Pharmingen™ PE rat anti-mouse CD8a) were added and incubated for 30 min at 25 ℃ in the dark. Red blood cells were lysed by adding lysis buffer (BD FACS™ lysing solution), centrifuged at 300 × g for 5 min, then the supernatant was discarded. The pellet was washed by adding 2 mL of PBS, centrifuged at 500 × g for 5 min, and then the supernatant was discarded. The pellet was resuspended in 500 µL PBS, and after mixing, the cells were detected by flow cytometry (Beckman, DXflex, USA) in accordance with the instrument’s instructions.

### Protective effect

For the protection studies, the mice were challenged with 100 μL of 5 × 10^5^ CFU/mL of WT 2308 *B. abortus* seven weeks after vaccination*.* Three weeks post-challenge, spleen weight, bacterial burden, CD4^+^ T cells, CD8^+^ T cells, IFN-γ, and IL-4 were measured as described above. Specific primers (P1: 5’—GAC GAA CGG AAT TTT TCC AAT CCC—3’, P2: 5’—TGC CGA TCA CTT AAG GGC CTT CAT—3’, P3: 5’—CCC CGG AAG ATA TGC TTC GAT CC—3’) were used to identify the spleen isolates of the mice post challenge. Primers P1 and P2 could identify *B. abortus*, with the PCR product fragment being 498 bp. Primers P2 and P3 could identify *B. abortus* strain 2308 with the PCR product fragment being 364 bp.

### Statistical analysis

The statistical analysis was conducted using the GraphPad Prism 8.0 software (GraphPad Software, CA, USA). The data for the spleen weight, CFU, antibodies, lymphocyte and cytokine are presented as the mean ± SD. The differences between groups were analyzed by a one-way analysis of variance t-test followed by Tukey’s honestly significant difference post test comparing all groups to one another.

## Supplementary Information

**Additional file 1.**

## Data Availability

The datasets generated and/or analysed during the current study are not publicly available due to the commercial interest of TECON, but are available from the corresponding author on reasonable request.

## Abbreviations

IFN-γ: Interferon-gamma
IgG: Immunoglobulin G
IL-4: Interleukin-4
IP: Intraperitoneal
WPC: Weeks post-challenge
wpi: Weeks post-infection
