# Supplementary material for: Combined immunization with inactivated vaccine reduces the dose of live B. abortus A19 vaccine
Source: BMC Vet Res. 2022 Apr 2;18:128. doi: 10.1186/s12917-022-03229-0 (PMC8976406; doi:10.1186/s12917-022-03229-0)
Supplement: Supplementary file 1 — Additional file 1. [file 12917_2022_3229_MOESM1_ESM.docx]

**Table 1 Mouse experimental data**

|  |  |  | Inactivated (I) | Low-dose Live + Inactivated (LI) | Low-dose Live (L) | High-dose Live (H) | Control (C) |
| --- | --- | --- | --- | --- | --- | --- | --- |
| Vaccinated | spleen weight | 7 | 0.198 ± 0.01^a^ | 0.223 ± 0.024^ab^ | 0.188 ± 0.01^a^ | 0.273 ± 0.03^b^ | 0.196 ± 0.011^a^ |
|  |  | 14 | 0.289 ± 0.023^ab^ | 0.252 ± 0.014^ac^ | 0.215 ± 0.015^cd^ | 0.3 ± 0.013^b^ | 0.195 ± 0.005^d^ |
|  |  | 21 | 0.251 ± 0.049^ab^ | 0.313 ± 0.064^ab^ | 0.242 ± 0.041^ab^ | 0.333 ± 0.024^a^ | 0.217 ± 0.012^b^ |
|  |  | 35 | 0.318 ± 0.012^ab^ | 0.449 ± 0.016^c^ | 0.335 ± 0.007^a^ | 0.526 ± 0.03^d^ | 0.275 ± 0.009^b^ |
|  |  | 49 | 0.415 ± 0.024^a^ | 0.568 ± 0.047^b^ | 0.399 ± 0.018^ac^ | 0.582 ± 0.032^b^ | 0.329 ± 0.011^c^ |
|  | spleen CFU | 7 | 0 ± 0^a^ | 5.214 ± 0.188^bc^ | 3.238 ± 0.094^d^ | 4.572 ± 1.144^cd^ | 0 ± 0^a^ |
|  |  | 14 | 0 ± 0^a^ | 5.324 ± 0.482^bc^ | 2.635 ± 0.273^d^ | 4.968 ± 0.309^c^ | 0 ± 0^a^ |
|  |  | 21 | 0 ± 0^a^ | 3.094 ± 0.949^b^ | 2.286 ± 0.278^bc^ | 3.952 ± 1.429^b^ | 0 ± 0^a^ |
|  |  | 35 | 0 ± 0 | 1.587 ± 1.016 | 0.334 ± 0.306 | 0.867 ± 0.809 | 0 ± 0 |
|  |  | 49 | 0 ± 0 | 1.279 ± 1.109 | 0.105 ± 0.181 | 0.771 ± 1.336 | 0 ± 0 |
|  | IgG | 7 | 0.405 ± 0.102^a^ | 0.48 ± 0.033^a^ | 0.439 ± 0.057^a^ | 0.603 ± 0.087^ab^ | 0.112 ± 0.017^c^ |
|  |  | 14 | 0.821 ± 0.097^a^ | 1.14 ± 0.06^b^ | 0.888 ± 0.061^a^ | 1.115 ± 0.109^b^ | 0.098 ± 0.004^c^ |
|  |  | 21 | 1.072 ± 0.111^a^ | 1.242 ± 0.053^a^ | 1.119 ± 0.058^a^ | 1.29 ± 0.062^ab^ | 0.088 ± 0.025^c^ |
|  |  | 35 | 1.111 ± 0.094^a^ | 1.326 ± 0.04^b^ | 1.178 ± 0.048^ab^ | 1.258 ± 0.055^ab^ | 0.056 ± 0.026^c^ |
|  |  | 49 | 0.827 ± 0.136^a^ | 1.281 ± 0.053^b^ | 0.87 ± 0.093^a^ | 1.099 ± 0.032^b^ | 0.051 ± 0.028^c^ |
|  | IFN-γ | 7 | 0.33 ± 0.128^a^ | 0.302 ± 0.078^ab^ | 0.216 ± 0.001^ab^ | 0.279 ± 0.02^ab^ | 0.134 ± 0.02^b^ |
|  |  | 14 | 0.36 ± 0.151^ab^ | 0.5 ± 0.04^a^ | 0.293 ± 0.049^ab^ | 0.281 ± 0.224^ab^ | 0.154 ± 0.033^b^ |
|  |  | 21 | 0.722 ± 0.113^a^ | 1.146 ± 0.064^b^ | 0.795 ± 0.021^a^ | 0.827 ± 0.094^a^ | 0.112 ± 0.013^c^ |
|  |  | 35 | 0.895 ± 0.125^a^ | 1.162 ± 0.164^a^ | 0.995 ± 0.125^a^ | 1.181 ± 0.068^a^ | 0.095 ± 0.012^b^ |
|  |  | 49 | 0.778 ± 0.079^a^ | 0.898 ± 0.022^b^ | 0.828 ± 0.031^ab^ | 0.916 ± 0.028^bc^ | 0.144 ± 0.011^d^ |
|  | IL-4 | 7 | 107.167 ± 4.274^ab^ | 147.667 ± 9.64^c^ | 81.1 ± 8.25^bd^ | 119.633 ± 17.879^ac^ | 61.1 ± 9.331^d^ |
|  |  | 14 | 82.733 ± 7.687 | 80.433 ± 3.139 | 75.833 ± 7.26 | 86.167 ± 7.366 | 70.5 ± 6.751 |
|  |  | 21 | 70.833 ± 4.167 | 76.667 ± 9.39 | 69.967 ± 5.064 | 67.6 ± 11.588 | 67.967 ± 3.47 |
|  |  | 35 | 64.933 ± 5.897^a^ | 79.167 ± 3.109^b^ | 68.4 ± 1.97^ab^ | 70.6 ± 2.691^ab^ | 64.067 ± 5.934^a^ |
|  |  | 49 | 68.733 ± 3.612 | 72.2 ± 3.676 | 64.167 ± 4.539 | 66.2 ± 9.65 | 64.167 ± 4.539 |
|  | T Cell | CD4^+^ | 18.65 ± 2.411^a^ | 26.08 ± 0.902^b^ | 19.217 ± 0.955^a^ | 27.227 ± 1.059^b^ | 11.307 ± 1.686^c^ |
|  |  | CD8^+^ | 5.513 ± 0.305^ab^ | 6.933 ± 1.309^bc^ | 5.08 ± 0.579^ab^ | 7.43 ± 0.52^c^ | 3.64 ± 0.139^a^ |
| Challenged | spleen weight | | 0.49 ± 0.027^a^ | 0.421 ± 0.026^b^ | 0.499 ± 0.023^a^ | 0.421 ± 0.033^b^ | 0.512 ± 0.026^a^ |
|  | spleen CFU | | 5.2 ± 0.384^a^ | 3.343 ± 0.46^b^ | 4.55 ± 0.386^a^ | 3.272 ± 0.554^b^ | 6.169 ± 0.725^c^ |
|  | WT | | 3.648 ± 0.549^a^ | 0 ± 0^b^ | 0 ± 0^b^ | 0 ± 0^b^ | 6.46 ± 0.729^c^ |
|  | IL-4 | | 80.77 ± 1.514^ab^ | 87.13 ± 3.139^bc^ | 73 ± 1^a^ | 92.87 ± 7.366^c^ | 72.73 ± 2.991^a^ |
|  | IFN-γ | | 1.279 ± 0.125^ab^ | 1.546 ± 0.164^a^ | 1.2 ± 0.1^b^ | 1.565 ± 0.068^a^ | 0.134 ± 0.02^c^ |
|  | CD4^+^ | | 20.42 ± 0.669^a^ | 25.43 ± 0.684^ab^ | 19.22 ± 0.955^a^ | 26.29 ± 0.569^b^ | 12.28 ± 0^c^ |
|  | CD8^+^ | | 5.98 ± 0.567^ab^ | 6.597 ± 1.196^bc^ | 5.08 ± 0.579^bc^ | 8.073 ± 0.642^c^ | 3.56 ± 0^b^ |

Note: For superscript notations, different letters mean significant difference (P < 0.05).
